# Hypermethylation of Interferon Regulatory Factor 8 (IRF8) Confers Risk to Vogt-Koyanagi-Harada Disease

**DOI:** 10.1038/s41598-017-01249-7

**Published:** 2017-04-21

**Authors:** Yiguo Qiu, Hongsong Yu, Yunyun Zhu, Zi Ye, Jing Deng, Wencheng Su, Qingfeng Cao, Gangxiang Yuan, Aize Kijlstra, Peizeng Yang

**Affiliations:** 1 The First Affiliated Hospital of Chongqing Medical University, Chongqing Key Laboratory of Ophthalmology, Chongqing Eye Institute, Chongqing, China; 2 University Eye Clinic Maastricht, Maastricht, The Netherlands

## Abstract

Aberrant methylation change of IRF8 confers risk to various tumors, and abnormal expression of IRF8 is involved in many autoimmune diseases, including ocular Behcet’s disease. However, whether the methylation change of IRF8 is associated with Vogt-Koyanagi-Harada (VKH) disease remains unknown. In the present study, we found a decreased IRF8 mRNA expression in association with a higher methylation level in monocyte-derived dendritic cells (DCs) from active VKH patients compared with the normal and inactive subjects. DCs incubated with cyclosporin a (CsA) or dexamethasone (DEX) showed a lower methylation and higher mRNA expression of IRF8 in active VKH patients. A demethylation reagent, 5-Aza-2′-deoxycytidine (DAC) showed a notable demethylation effect as evidenced by increasing the mRNA expression and reducing the methylation level of IRF8. It also suppressed the Th1 and Th17 responses through down-regulating the expression of co-stimulatory molecules (CD86, CD80, CD40), and reducing the production of pro-inflammatory cytokines (IL-6, IL-1β, IL-23, IL-12) produced by DCs. These findings shows that hypermethylation of IRF8 in DCs confers risk to VKH disease. Demethylation of IRF8 may offer a novel therapeutic strategy protect against VKH disease.

## Introduction

VKH disease is an autoimmune disorder which is distinguished by granulomatous panuveitis in association with multisystemic involvement. It is a sight-threatening disease and is one of the top common uveitis type in China^1, 2^. Although the exact pathogenesis of VKH disease is poorly understood, an autoimmune response against melanocytes has been proposed^3^. Moreover, accumulating evidence has demonstrated a hyperactive response of Th1 and Th17 cells, suggesting these T cell subsets are vital in the pathogenesis of VKH disease^4, 5^.

Dendritic cells (DCs) belong to the professional antigen presenting cells (APCs) and serve as important mediators in inducing primary immune reactions, initiating adaptive immune responses, as well as generating immune tolerance^6, 7^. DCs are a vital link between the innate and adaptive immune responses. It is well established that T cells can not be fully activated without the roles played by DCs. Upon recognizing pathogens via pattern recognition receptors, the surface markers of DCs are up-regulated and a battery of pro-inflammatory cytokines are produced to prompt naïve T cells to differentiate into different subsets, and consequently leading to an immune response^8, 9^. A number of autoimmune and inflammatory diseases, including uveitis, are caused by an abnormal immune response by virtue of differently activated T cell subsets which are determined by DCs^6^. Previous study from our group also shows that therapeutic treatment which affects the function of DCs can inhibit Th17 responses in VKH disease^10^.

Interferon regulatory factor 8 (IRF8) belongs to the IRF transcription factor family^11^, that modulates the immune response and other important physiologic processes, including cell growth and oncogenesis^12^. It is also an important regulator for the function of several immune cells, including DCs^13, 14^. The development and activation of DCs are closely regulated by IRF8^15, 16^ and lack of IRF8 results in the impairment of DC function during inflammation^17^. Moreover, IRF8 polymorphisms have been found to be involved in various autoimmune diseases, including ocular Behcet’s disease^18–21^.

Studies in the experimental autoimmune uveitis model in mice showed that intraocular inflammation is exacerbated in mice with a targeted IRF8 deletion in their T cells whereby disease enhancement correlated with Th17 cell expansion and a decrease in T regulatory cells^22^. These studies prompted us to further investigate the possible role of IRF8 in clinical uveitis whereby we decided to investigate whether the epigenetic control of IRF8 function might be associated with disease development. VKH disease was chosen as an autoimmune uveitis entity, whereby we focused on DNA methylation as a possible mechanism controlling the function of IRF8.

DNA methylation is a key mechanism for epigenetic regulation of gene expression. Hypermethylation is often associated with gene silencing^23^. Aberrant DNA methylation of gene promoters is a commonly observed epigenetic change in various cancers^24^. Recent studies show that IRF8 exerts anti-tumor responses by modulating immune responses^25^, suggesting that the methylation change of IRF8 may affect immune responses. However, whether aberrant methylation of IRF8 is associated with autoimmune diseases remains unknown. In our study, the methylation level and mRNA expression of IRF8 in DCs obtained from VKH patients were investigated. In addition, we further studied the demethylation effect of IRF8 on DCs’ maturation and function. The results show that hypermethylation of IRF8 confers risk to VKH disease.

## Results

### Decreased IRF8 mRNA level and up-regulated methylation level of the IRF8 promoter were noted in DCs from active VKH patients

**Figure 1:**
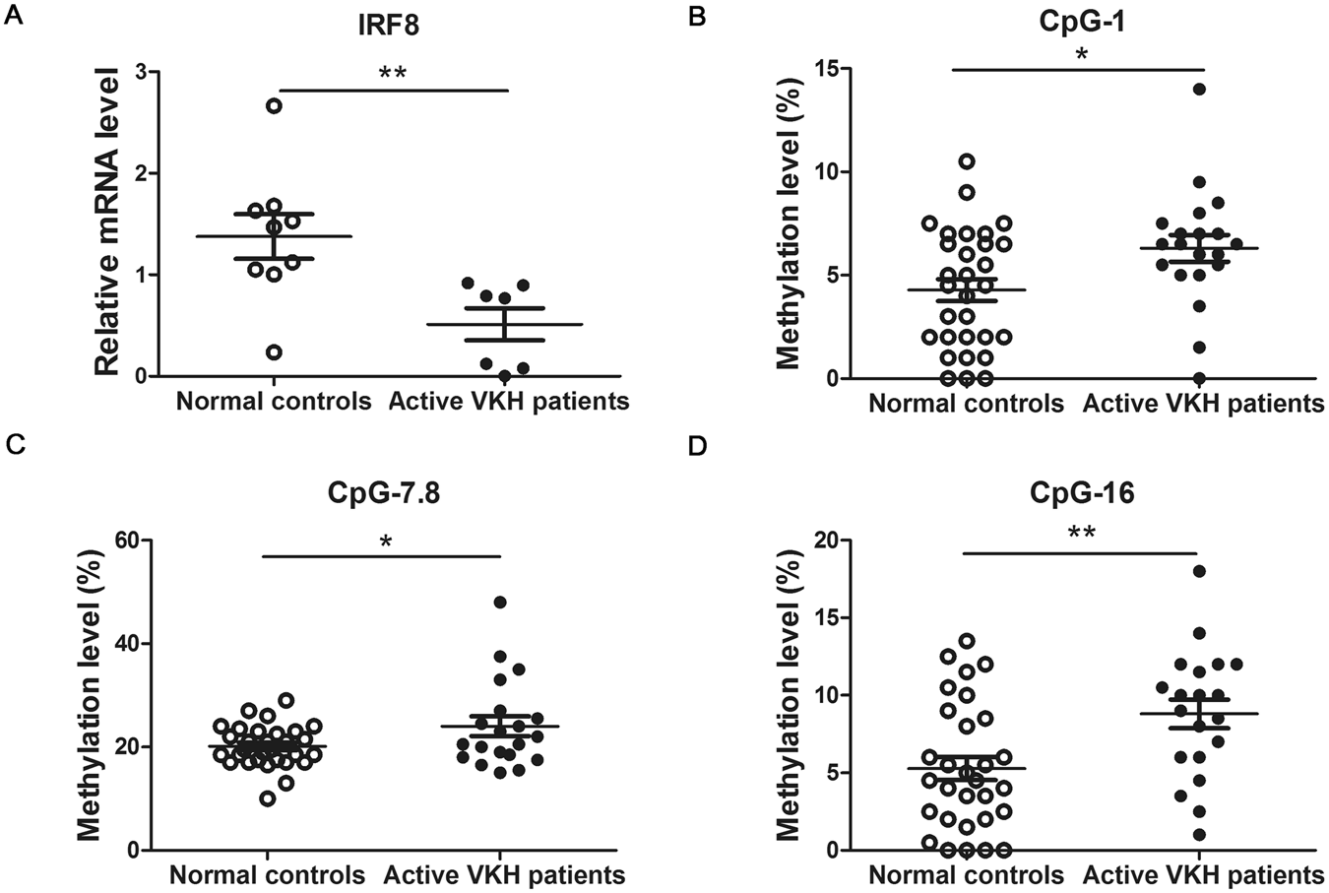
A lower mRNA expression and a higher methylation level of IRF8 in the DCs of active VKH patients is detected than normal controls. (**A**) DCs from normal controls and active VKH patients were cultured and collected to detect the expression of IRF8 with real-time PCR (normal controls: n = 9; VKH patients: n = 7, **p < 0.01). Methylation levels of the CpG sites between −441 and −225 from TSS of the first exon were detected with matrix assisted laser desorption ionization time-of-flight (MALDI-TOF) mass spectrometer in the DCs of VKH patients and normal controls. The methylation changes shown in CpG_1 (**B**), CpG_7.8 (**C**) and CpG_16 (**D**) of VKH patients were increased compared with normal controls (VKH, n = 20; normal controls, n = 30, *p < 0.05, **p < 0.01). The data are shown as mean ± SEM. All the values were normally distributed. Unpaired t test was used to compare the methylation change of a certain CpG site between two groups.

IRF8 participates in several autoimmune disease^18–20, 26^. However, whether IRF8 is associated with uveitis is poorly studied. Therefore, we investigated the mRNA level of IRF8 by real-time PCR in DCs from VKH and normal subjects. We found the mRNA level of IRF8 was remarkably decreased in active VKH subjects compared to controls (Fig. 1A, **p < 0.01). Human IRF8 gene is in the position of pos. 84,490,013 to 84,490,351 on chromosome 16^27^. Positions of the CpGs between −441 and −225 from TSS of the first exon were analyzed. We were able to detect 9 CpG sites. Hypermethylation of CpG_1, CpG_7.8 and CpG_16 was observed in the DCs of active VKH patients compared with the normal controls (Table 1, Fig. 1B,C,D, *p < 0.05, **p < 0.01).

**Table 1:** Methylation levels of IRF8 promoter in DCs from VKH patients versus normal controls.

CpG site	VKH Methylation level (%, mean ± SD)	NC Methylation level (%, mean ± SD)	p value
CpG_1	6.30 ± 2.86	4.28 ± 2.88	0.0189*
CpG_3	5.75 ± 1.52	5.68 ± 1.99	0.8994
CpG_4, 5, 6	6.29 ± 2.45	5.43 ± 4.45	0.4474
CpG_7,8	24.35 ± 9.16	20.10 ± 4.00	0.0321*
CpG_9,10	10.85 ± 3.39	11.65 ± 3.69	0.4517
CpG_11,12,13	7.29 ± 2.59	7.33 ± 2.92	0.8624
CpG_14	3.23 ± 2.86	2.82 ± 1.90	0.5598
CpG_15	5.60 ± 9.09	1.96 ± 3.89	0.0727
CpG_16	8.80 ± 4.13	5.32 ± 4.02	0.0044*

VKH patients (n = 20, M:F = 14:6); Age (years: mean ± SD): 38.85 ± 11.47.

Normal control (n = 30, M:F = 21:9); Age (years: mean ± SD): 38.99 ± 11.04.

### Down-regulated methylation level and restored mRNA expression of IRF8 were observed in DCs from inactive VKH patients

**Figure 2:**
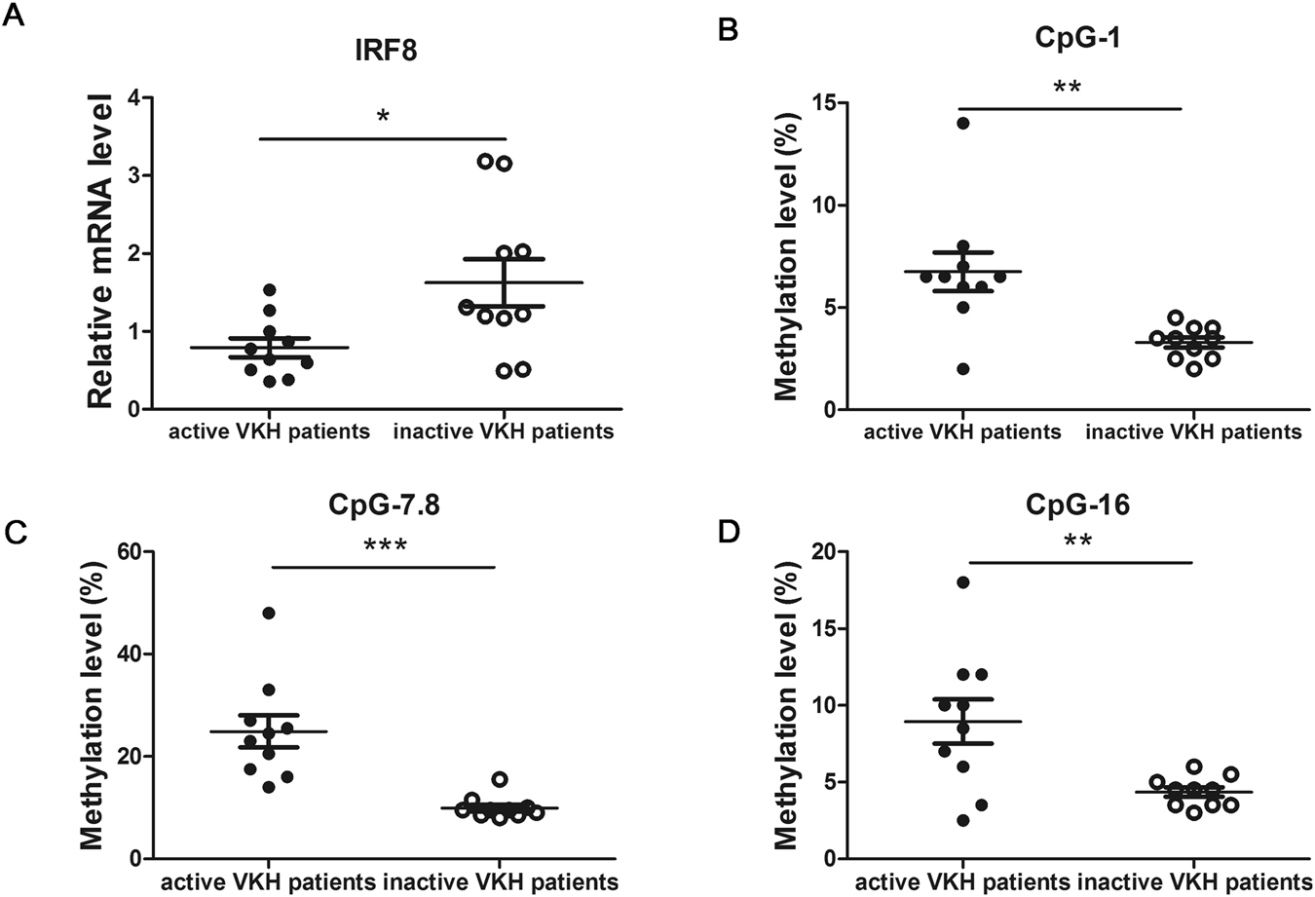
The methylation and the mRNA expression changes of IRF8 affect the activity of VKH patients. (**A**) DCs from inactive and active VKH patients were cultured and collected to detect the expression of IRF8 with real-time PCR (n = 10, *p < 0.05). The methylation changes shown in CpG_1 (**B**), CpG_7.8 (**C**) and CpG_16 (**D**) of inactive VKH patients were notably lower than active patients (n = 10, **p < 0.01, ***p < 0.001). The data are shown as mean ± SEM. Unpaired t test was used to compare the mRNA level and methylation changes between two groups.

Up-regulated methylation and decreased mRNA expression of IRF8 were noted in active VKH patients not receiving medication. Cyclosporin a and steroids are the most commonly prescribed drugs for VKH patients^2^. Nevertheless, how these conventional drugs used for the treatment of VKH disease affect the methylation change and expression of IRF8 remains unknown. Therefore, we investigated the methylation level and mRNA expression of IRF8 in DCs from inactive VKH patients that had previously received drugs. We found that the mRNA level of IRF8 was restored in the inactive VKH patients following treatment compared with active patients (Fig. 2A, *p < 0.05). Additionally, the methylation changes of CpG_1, CpG_7.8 and CpG_16 were decreased in the inactive VKH subjects compared to the active subjects (Fig. 2B,C,D, **p < 0.01, ***p < 0.001).

### CsA and DEX reduced the methylation level and restored the mRNA expression of IRF8 in DCs from active VKH patients

**Figure 3:**
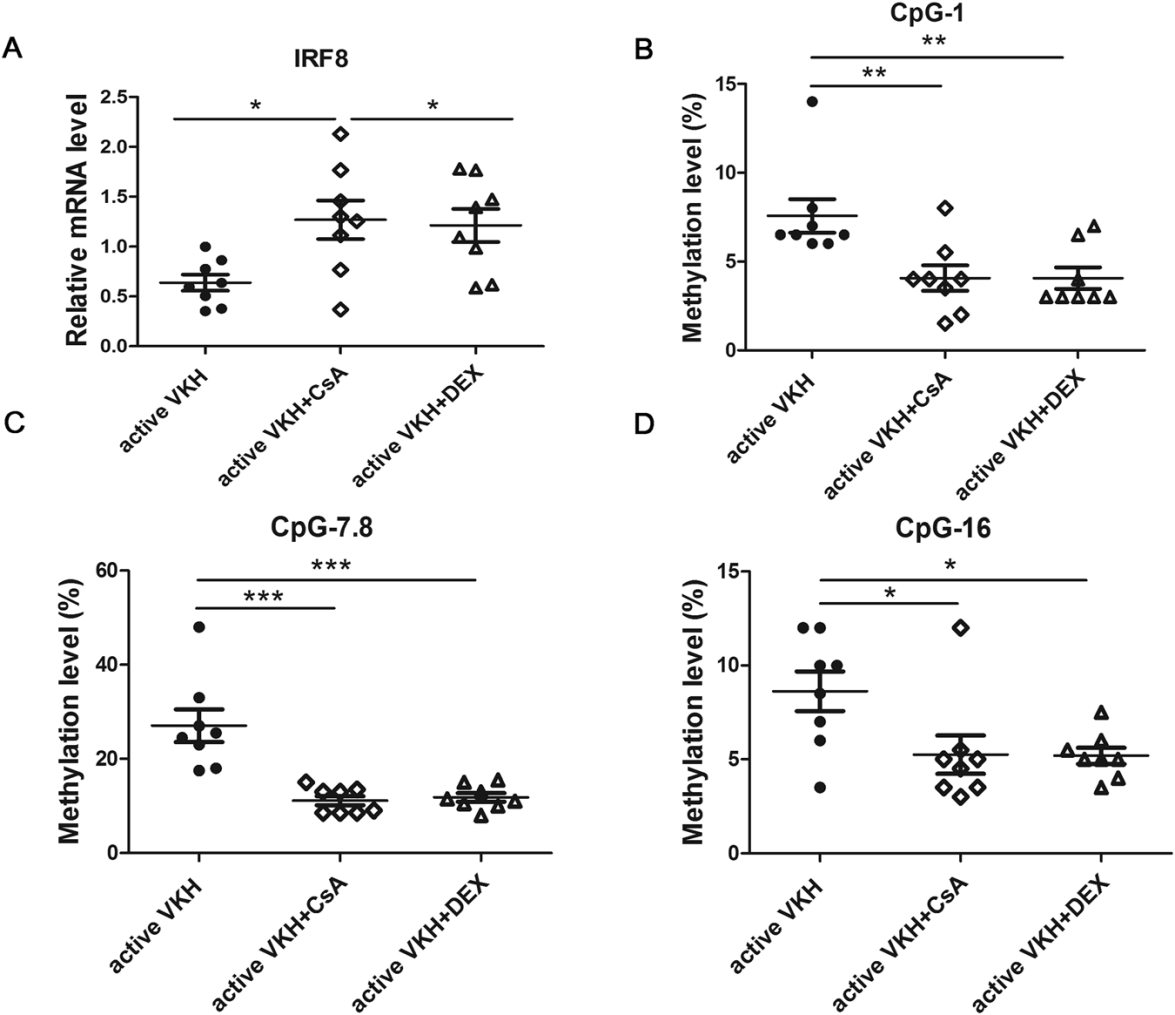
CsA and DEX treatment affects the methylation change and mRNA expression of IRF8 in active VKH patients. (**A**) The mRNA expression of IRF8 was detected by real-time PCR in DCs with or without the treatment with CsA or DEX. DCs were obtained from active VKH patients (n = 8, *p < 0.05). The methylation changes shown in CpG_1 (**B**), CpG_7.8 (**C**) and CpG_16 (**D**) of CsA treated and DEX treated DCs were significantly decreased compared to the untreated DCs from active VKH patients (n = 8, *p < 0.05, **p < 0.01, ***p < 0.001). The data are shown as mean ± SEM. One way-ANOVA, followed by Bonferroni correction was used to compare the mRNA level and methylation changes among multiple groups.

CsA and corticosteroids, such as DEX are conventional drugs used for treating several autoimmune diseases, including VKH disease. We found that the drug treatment affected the activity of VKH disease, and the methylation level of IRF8 showed significant differences between active and inactive patients (Fig. 2). Based on the aforementioned results, we further confirmed whether CsA and DEX affect the activity of disease by regulating the methylation status of IRF8. The methylation change and mRNA expression of IRF8 in DCs treated with 50 ng/ml CsA or 100 ng/ml DEX were measured. We found that treatment with CsA or DEX, resulted in an increase of IRF8 mRNA expression (Fig. 3A, *p < 0.05). On the other hand, the methylation levels of CpG_1, CpG_7.8 and CpG_16 were remarkably decreased compared with that seen in untreated DCs obtained from active VKH patients (Fig. 3B,C,D, *p < 0.05, **p < 0.01, ***p < 0.001).

### DAC increased the mRNA expression of IRF8 and reduced the methylation level of IRF8 promoter in active VKH subjects

**Figure 4:**
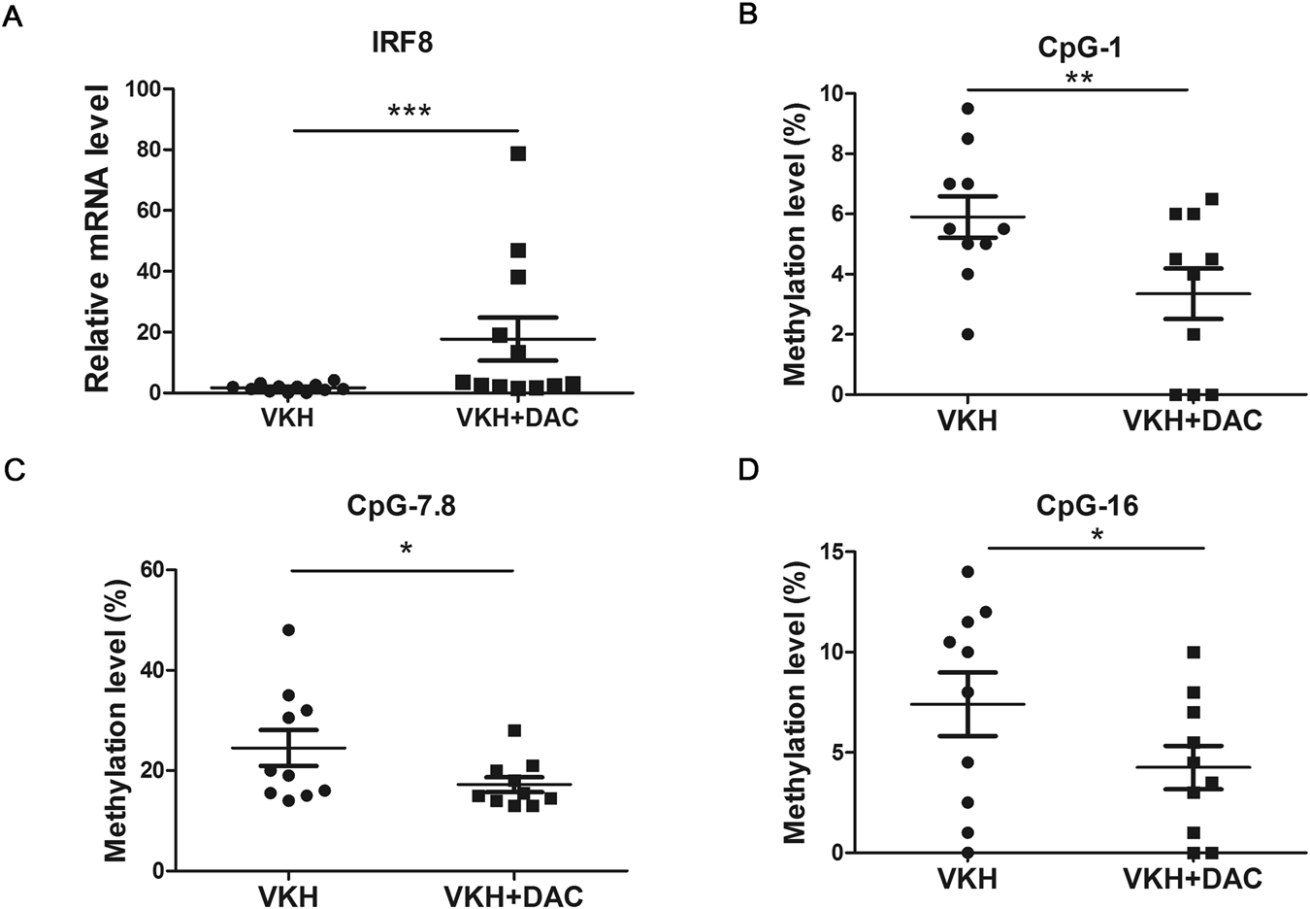
DAC treatment shows a demethylation effect and restores the mRNA expression of IRF8. (**A**) DAC treated and untreated DCs from active VKH patients were collected to detect the expression of IRF8 with real-time PCR (VKH, n = 12, VKH + DAC, n = 12, ***p < 0.001). The methylation changes in CpG_1 (**B**), CpG_7.8 (**C**) and CpG_16 (**D**) of DAC treated and untreated DCs from VKH patients were evaluated with MALDI-TOF mass spectrometer (VKH, n = 10; VKH + DAC, n = 10, *p < 0.05, **p < 0.01). The data are shown as mean ± SEM. Paired-t test was used to compare the methylation changes between two groups.

To investigated the demethylation effect of the IRF8 promoter region, a DNA demethylation reagent, DAC, was added to DC cultures. The results showed a significant increase of IRF8 mRNA expression with the treatment of DAC compared with the DCs without DAC treatment (Fig. 4A, ***p < 0.001). Correspondingly, the methylation changes of CpG_1, CpG_7.8 and CpG_16 of DAC-treated cells were also reduced compared to untreated DCs (Fig. 4B,C,D, *p < 0.05, **p < 0.01).

### DAC diminished surface markers expression of DCs obtained from active VKH patients

**Figure 5:**
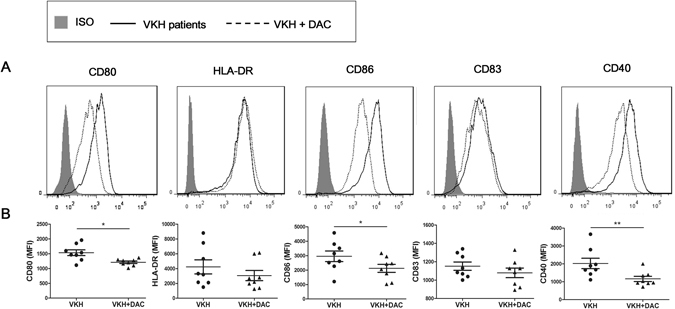
DAC reduces the expression of surface markers of DCs from VKH patients. Immature DCs from VKH patients were cultured with or without the presence of DAC for 6 days, then followed by stimulation with 100 ng/mL LPS for 24 hours. DCs were subsequently harvested for flow cytometry analysis with specific surface antibodies against CD80, HLA-DR, CD86, CD83 and CD40. (**A**) Histograms with overlays are from representative experiments of DAC treated and untreated DCs from VKH patients. (**B**) Mean fluorescence intensity (MFI) of surface markers on DAC treated and untreated DCs from VKH patients. Data are shown as mean ± SEM. Statistical analysis was performed using the paired-samples t-test (n = 8, *p < 0.05; **p < 0.01).

DCs are obligatory in initiating and maintaining immune responses. The co-stimulatory molecules and surface markers uniquely expressed by DCs facilitate the activation of naïve T cells and promote the primary immune response^28^. The antigen presentation of DCs and the differentiation of Th cell subsets are closely relied on co-stimulatory molecules^29^. For investigating the demethylation effect of IRF8 on DCs’ maturation, we examined the surface markers CD86, CD83, CD80, CD40 and HLA-DR in DAC treated and untreated DCs from VKH patients by flow cytometry. The expression of CD80, CD86 and CD40 was notably down-regulated following DAC treatment (Fig. 5, *p < 0.05, **p < 0.01), suggesting that demethylation of IRF8 might negatively affect DC function, leading to an inhibition of the differentiation of effector T cells.

### DAC suppressed the production of inflammatory cytokines by DCs from active VKH patients

**Figure 6:**
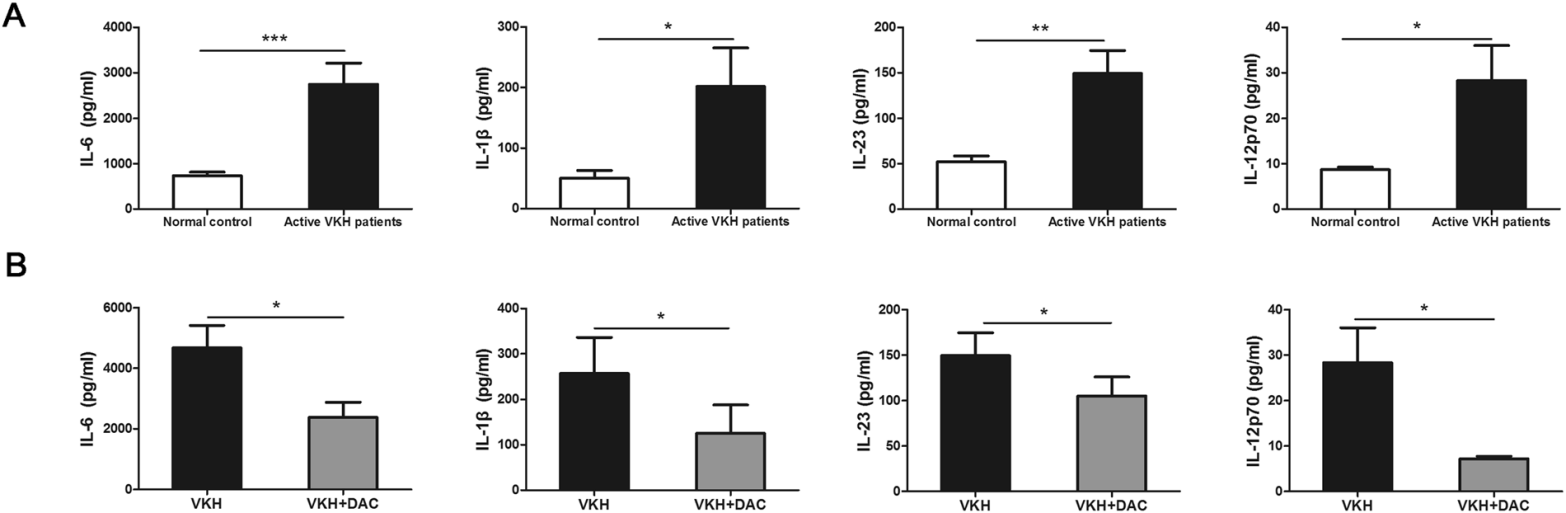
DAC affects the production of inflammatory cytokines by DCs from VKH patients. CD14^+^ monocytes from VKH patients and normal controls were cultured for 6 days and then stimulated with 100 ng/ml LPS for 24 hours. The protein concentrations of IL-6, IL-1β, IL-23 and IL-12p70 in the supernatants were determined by ELISA. (**A**) The expression of IL-6, IL-1β, IL-23 and IL-12p70 was significantly increased in VKH patients compared with normal controls (n = 12, *p < 0.05,**p < 0.01, ***p < 0.001). (**B**) The protein concentrations of IL-6, IL-1β, IL-23 and IL-12p70 were reduced if cells (from VKH patients) were pretreated with 10 µM of DAC (n = 12, *p < 0.05). The data are shown as mean ± SEM. The paired-samples t-test was performed for the statistical analysis.

DCs is capable to differentiate naïve T cells into different Th cell subsets depending on the type of secreted cytokines^28^. Given that Th1 and Th17 cells are important effector T cells in the pathological process of VKH disease^4, 5^, we studied whether DAC affects cytokine production which facilitate either Th1 or Th17 responses. The Th17-promoting cytokines IL-6, IL-1β, and IL-23 from VKH patients was approximately 3.7-, 4.0- and 2.9-fold higher than observed in the normal controls. Similarly, the Th1-promoting cytokine IL-12p70 was 3.3-fold higher than in the normal controls (Fig. 6A, *p < 0.05,** p < 0.01, ***p < 0.001). However, DAC treatment resulted in a reduction of IL-6, IL-1β, IL-23 and IL-12p70 by approximately 2.0-, 2.1-, 1.4- and 3.9-fold (Fig. 6B, *p < 0.05). The fold changes of protein between the VKH group and controls as well as the effect in VKH following DAC treatment are shown in Supplementary Table S1.

### DAC treatment suppressed DC-mediated Th1/Th17 responses in CD4^+^ T cells

**Figure 7:**
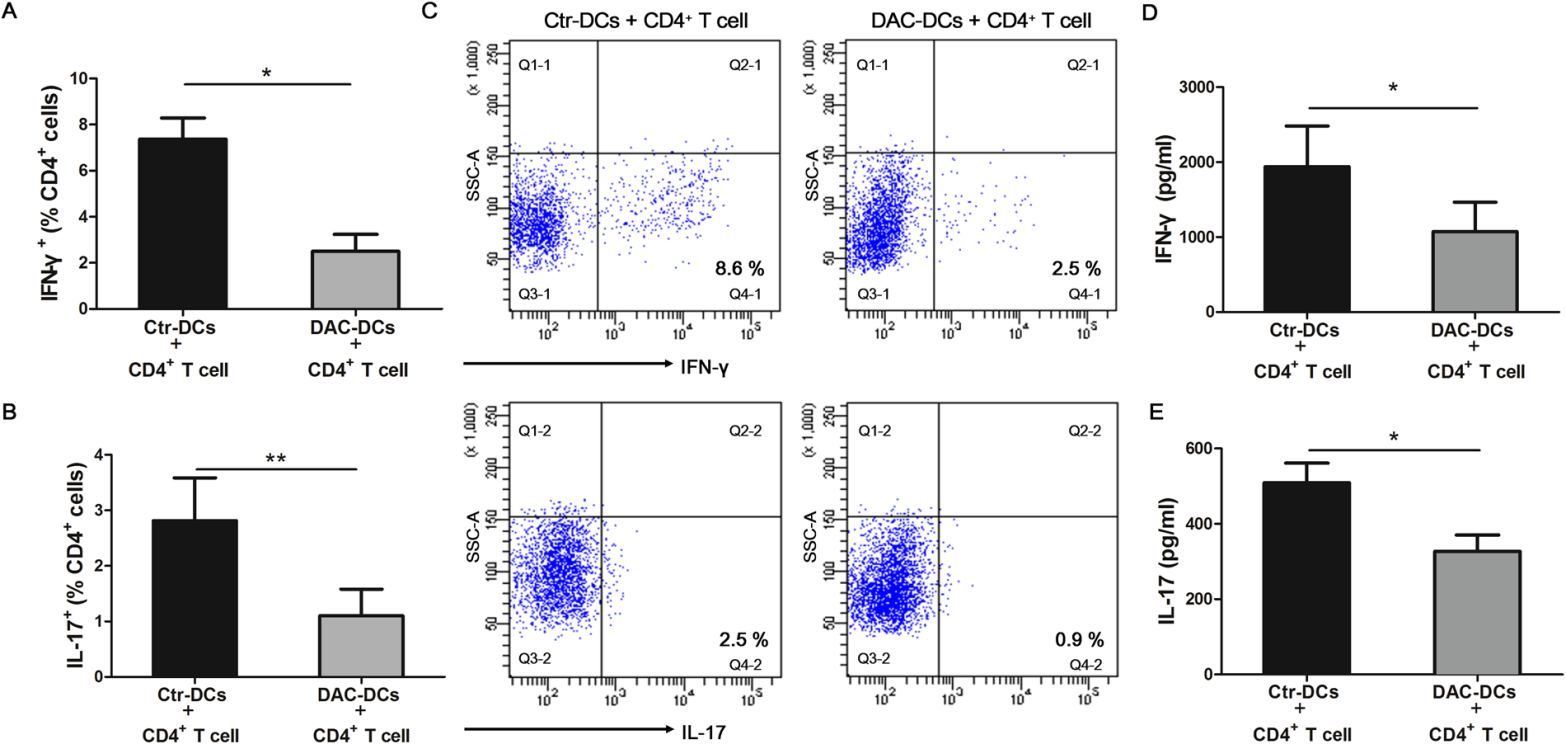
DAC treatment inhibits the Th1 and Th17 responses. Allogeneic CD4^+^ T cells (obtained from healthy controls) were co-cultured with DAC-treated or –untreated DCs (derived from VKH patients) for 5 days. The frequencies of IFN-γ^+^ (**A**) and IL-17^+^ cells (**B**) in the CD4^+^ T cells co-cultured with DAC-treated or –untreated DCs were evaluated by flow cytometry (n = 7, *p < 0.05,**p < 0.01). (**C**) The representative results of the IFN-γ^+^ and IL-17^+^ cells in the CD4^+^ T cells co-cultured with DAC-treated or –untreated DCs. The protein levels of IFN-γ (**D**) and IL-17 (**E**) in the cell culture supernatants were measured by ELISA (n = 7, *p < 0.05). The data are shown as mean ± SEM. Ctr-DCs: control DCs (DAC-untreated DCs), DAC-DCs: DAC-treated DCs. Paired t test was used to compare the protein concentrations of IFN-γ and IL-17 and the frequencies of IFN-γ^+^ and IL-17^+^ cells between two groups.

It is generally accepted that DCs are not only critical in activating T cells, but also important in regulating the differentiation of Th cell subsets^30, 31^. To investigate the role of DAC-treated DCs in the Th1 and Th17 response, we co-cultured DAC-treated or untreated-DCs with CD4^+^ T cells for 5 days, whereby T cells were obtained from normal controls and DCs were obtained from VKH patients, as previously described^32^. Our data revealed that the frequencies of CD4^+^IFN-γ^+^ and CD4^+^IL-17^+^ cells were significantly lower in T cells co-cultured with DAC-treated DCs than the cells which were co-cultured with untreated DCs (control DCs) (Fig. 7A,B,C, *p < 0.05, **P < 0.01). In parallel, the CD4^+^ T cells which co-cultured with DAC-treated DCs produced much less IFN-γ or IL-17 than the cells cultured with-untreated DCs (Fig. 7D,E, *p < 0.05).

## Discussion

This study shows that the IRF8 mRNA level in DCs acquired from active VKH subjects is lower compared to normal controls, whereas the methylation of the IRF8 promoter region is higher. Testing DCs from patients that had responded to treatment and who had entered an inactive phase of the disease showed a down-regulated methylation level and increased mRNA expression of IRF8 compared to active VKH patients. The role of drug treatment was confirmed by *in vitro* experiments that showed a lower methylation and higher mRNA expression of IRF8 when DCs from active untreated VKH patients were incubated with CsA or DEX. Although a previous study did not find any association of IRF8 gene polymorphisms with VKH disease^21^, our results revealed IRF8 methylation changes in this patient group which strongly suggests that it is involved in the pathogenesis of this particular uveitis entity. Our findings support earlier observations showing the important role of IRF8 in the control of experimental autoimmune uveitis (EAU) mice model^22^.

Although the exact etiology is poorly studied, a susceptible genetic background may predispose individuals to VKH disease. A assured link between the human leukocyte antigens (HLA)-DRw53/HLA-DR4 and VKH disease has been reported in Han Chinese^33^. Moreover, our group performed a genome-wide association study (GWAS) which not only proved the HLA gene closely related to VKH disease, but also found a locus in HLA-DRB1/DQA1 that was strongly associated with VKH disease^34^. VKH patients with different HLA types may have a different IRF8 methylation status, and HLA type may affect the response to *in vitro* demethylation studies. Further in-depth studies are needed to address this issue.

Cyclosporin and steroids are the most commonly prescribed drugs for VKH patients^2^. In the present study, we strictly enrolled inactive patients who had just finished a treatment period with CsA and steroids, and found the IRF8 methylation level was significantly diminished in these inactive subjects. We therefore investigated whether these drugs affect the activity of disease by influencing the methylation level of IRF8 in a series of *in vitro* experiments. We focused on these drugs although other agents that are often used in the treatment of clinical uveitis such as alkylating agents (cyclophosphamide etc.), antimetabolites (azathioprine, MTX) and anti-TNF antibody, may also have an effect on IRF8 methylation. Further studies on the effect of other drugs on IRF8 methylation are needed to investigate whether the efficacy of these drugs might also involve the IRF8 pathway.

To investigate the functional consequences of IRF8 methylation on DC function we performed a series of experiments with a methylation inhibitor. We first focused on surface markers expressed on DCs since they promote TCR signaling and result in the activation and differentiation of T cells^35, 36^. We found DAC treatment significantly reduced CD86, CD80 and CD40, indicating that the methylation changes can regulate the maturation of DCs. We subsequently addressed the effect of methylation on the cytokines secreted by DCs since they create the microenvironment dictating Th subtype differentiation. In agreement with earlier study, a significant increase of IL-6, IL-1β, IL-23 and IL-12p70 protein concentrations in the DCs of active VKH subjects compared to healthy subjects was noted^2^. We do not believe that the observed effects might be explained by DAC induced toxicity since a previous study showed that treating human multiple myeloma cell lines with the same 10 μM DAC as used in our study, could restore the mRNA expression of IRF8 without showing toxicity^27^. However, our findings are in disagreement with earlier reports in mice, whereby IRF8 seemed to be a positive regulator of inflammation, as shown by experiments whereby IL-12 production was stimulated by IRF8 in murine DCs^37^. This discrepancy may be caused by a different way that IRF8 acts in mice compared to humans. Although IRF8 promoted the generating of pro-inflammatory factors in murine DCs, it exerted negative regulation effects on IL-6 generating in human DCs^38^. Taken together, species/cell type-specific functions of IRF8 may exist. The possible mechanism of which IRF8 acts differently in different cell types needs to be explored in the future.

Furthermore, we investigated whether IRF8 methylation in DCs could affect the Th1 and Th17 responses. IRF8 was demonstrated to be a transcriptional inhibitor for the differentiation of Th17 cell^39^. Moreover, a study from our team found that up-regulation of IRF8 mediated a suppression effect of rIL-27 on Th17 cell differentiation in human^40^. In agreement with these earlier studies, we now showed that treatment with DAC, increased the expression of IRF8 which was associated with an altered DC function which in turn suppressed the Th17 responses. Importantly, our data also showed that IRF8 expression in DCs may negatively regulate Th1 cell responses in VKH, as shown by the inhibit effects of DAC-treated DCs on the percentages of CD4^+^ IFN-γ.

However, it is essential to recognize that there are still limitations in our study. Firstly, we have analyzed the methylation changes of IRF8 in a relatively small sample and further longitudinal studies in a cohort of VKH patients are needed to confirm our findings. Secondly, similar to previous studies^41, 42^, there is a technical limitation to evaluate the methylation changes using the MassARRAY system, since only a limited region of the promoter can be covered. Moreover, the changes observed in IRF8 may coincide with one or more inflammatory factors operative in the frame of VKH disease pathogenesis and a more (genome wide) systematic approach is necessary to improve our understanding of the pathology of this disease. Further studies in bigger cohorts and using a more comprehensive detection method, such as epigenome-wide association studies (EWAS) are required to validate the relationship between the aberrant methylation status with disease susceptibility, activity and treatment response. Additionally, there is a lack in methods to regulate specific methylation changes in a certain gene. We used a generally accepted *in vitro* method to regulate the methylation status using the demethylation reagent, DAC. Whether the observed changes can only be ascribed to IRF8 promoter methylation or whether methylation of other genes also plays an important role in our *in vitro* model needs further study.

In summary, the present study shows that hypermethylation of IRF8 in DCs confers risk to VKH disease. The beneficial effect of conventional therapy (CsA and DEX) on disease activity in VKH may be related to methylation changes of IRF8 mediated by these drugs. Demethylation with DAC reveals beneficial effects by regulating the function of DCs, and subsequently inhibiting pro-inflammatory Th1/Th17 responses. Targeting methylation of IRF8 gene promoter sites may provide a promising therapeutic target protect against VKH disease.

## Materials and Methods

### Subjects

Forty-seven active VKH subjects (31 male and 16 female, average age: 37.22 ± 12.98 years old), 10 inactive VKH subjects (6 male and 4 female, average age: 36.00 ± 9.92 years old) and a total of 56 healthy subjects (36 male and 20 female, average age: 39.27 ± 11.15 years old) were enrolled for this study. VKH disease was diagnosed according to the international committee revised VKH diagnostic criteria^43^. All active VKH subjects enrolled in this study show typical ocular inflammation signs. The extraocular symptoms included tinnitus (40.4%) and dysacusis (36.2%), alopecia (44.7%), poliosis (38.3%) and vitiligo (31.9%). All enrolled patients were diagnosed in our clinic uveitis center during December, 2015 to October, 2016. Patients with active ocular inflammation were either not treated or stopped using the drugs more than one week before blood sampling. The inactive patients had received treatment with conventional drugs before sampling, as shown in Supplementary Table S2. Our study strictly complied with the Declaration of Helsinki. Written informed consents for all enrolled subjects were provided. The study was approved by the Clinical Ethical Research Committee of Chongqing Medical University.

### Cell culture

CD14 and CD4 mAb-conjugated magnetic microbeads (Miltenyi Biotec, Germany) were used to isolate CD14^+^ monocytes and CD4^+^ T cells from VKH and the healthy subjects (purity >90%). The RPMI 1640 medium with 10% fetal bovine serum and 100 U/mL penicillin/streptomycin was used to culture the cells. To generate the monocyte-derived DCs, CD14^+^ monocytes were stimulated with 100 ng/ml granulocyte macrophage colony stimulating factor (GM-CSF, Acro Biosystems, Newark, DE, USA) and 50 ng/ml interleukin-4 (IL-4, Acro Biosystems, Newark, DE, USA) for 6 days. Half of the medium was then refreshed at the 4^th^ day. Mature DCs were subsequently generated by stimulation with 100 ng/ml lipopolysaccharide (LPS) for 24 hours. The co-culture experiment was performed as previously described^32^. Briefly, the DCs were co-cultured with CD4^+^ T cells (DC: T cell ratio = 1:5) with or without DAC for 5 days. The flow cytometry tests were performed to detect the intracellular IFN-γ and IL-17. The culture supernatants were used for ELISA assay.

### *In vitro* drug treatment

To investigate the DNA demethylation effect, DCs were cultured with or without 5-Aza-2′-deoxycytidine (DAC, Sigma-Aldrich, USA) at the concentration of 10 μM^27^ for 6 days, then incubated with 100 ng/ml LPS for additional 1 day. Cells and supernatants were then harvested for further assays.

To study the effect of CsA (Ruibang Pharmaceuticals, Zhejiang, China) and dexamethasone (DEX) (Sigma-Aldrich, USA) on the methylation changes, DCs from the active VKH patients were incubated with 50 ng/ml CsA or 100 ng/ml DEX as previously described^44^ for 6 days, then stimulated with 100 ng/ml LPS for 1 day.

### Real-time PCR analysis

TRIzol reagent (Invitrogen, CA, USA) was used to extract the total RNA from DCs under the manufacturer’s instructions. Complementary DNA was reverse transcribed by the PrimeScript RT kit (Takara, Dalian, China). SYBR Premix Ex TaqTM II (Takara, Dalian, China) was used to perform PCR analysis for IRF8 with the ABI Prism 7500 system (Applied Biosystems, CA, USA). The conditions were 95 °C for 10 minutes, 95 °C for 15 seconds and repeated for 40 cycles, then 60 seconds at 60 °C. Relative quantification was analyzed with 2^−ΔΔCt^ method as described previously^45^. The sequences of primers were as follows: IRF8: forward: 5′-GAAGACGAGGGTTACGCTGTG-3′, reverse: 5′-TCCTCAGGAACAATTCGGTAA-3′. β-actin: forward: 5′-ACTGGAACGGTGAAGGTGACAG-3′, reverse: 5′-GGTGGCTTTTAGGATGGCAAG-3′.

### DNA extraction and bisulfite treatment

The QIAamp DNA Kit (Qiagen, CA, USA) was used to extract the total DNA according to the manufacturer’s instruction. EZ DNA Methylation Kit (Zymo Research, CA, USA) was used to perform the bisulfite conversion of genomic DNA (800 ng). During the conversion process, the following cycling conditions were repeated for 20 cycles: 95 °C for 30 seconds, followed by 50 °C for 15 minutes.

### Quantitative methylation analysis of the IRF8 by Sequenom MassARRAY EpiTYPER System

DNA methylation level of the IRF8 promoter was quantified by using Sequenom MassARRAY EpiTYPER analysis using the following procedure: the initial denaturation at 95 °C for 4 minutes, denaturation at 95 °C lasts 20 seconds for 45 cycles, then annealing at 58 °C for 30 seconds, extension at 72 °C for 1 minute, and then incubation at 72 °C for 3 minutes. The primers employed for IRF8 methylation analysis were designed for 195 bp of the MassARRAY® EpiTYPER (Sequenom, Inc., CA, USA). The analyzed region of IRF8 is located −441 bp to −225 bp up-stream from the transcription initiation site (TSS) of the first exon. Methylation primer of IRF8 was as shown: forward: 5′-aggaagagagGGGTAGTTAGTTTTTGGTTGTGGAT-3′; reverse: 5′-cagtaatacgactcactatagggagaaggctTACAAAAAAACTTTCCCAAAAATTC-3′. The shrimp alkaline phosphatase (SAP) and MassCLEAVE reaction were then performed. The end-products were desalted and dispensed to a SpectroCHIP, using a MassARRAY™ Nanodispenser (Sequenom, Inc., CA, USA). A MassARRAY compact matrix assisted laser desorption ionization time-of-flight (MALDI-TOF) mass spectrometer (Sequenom, Inc., CA, USA) was used to acquire the spectra. The methylation results were analyzed with EpiTyper software (Sequenom, Inc., CA, USA).

### Flow cytometry

DAC-treated and –untreated DCs were incubated with anti-human HLA-DR- phycoerythrin-cyanin 5, CD86-allophycocyanin, CD80-phycoerythrin, CD40-peridinin chlorophyll-cyanin 5.5 and CD83-phycoerythrin at 4 °C for 30 minutes. All these antibodies were bought from BioLegend (CA, USA). Flow cytometry (FACSAira, BD Biosciences, USA) tests were performed to measure the changes of the markers. The results were shown as median fluorescence intensity (MFI) and were analyzed by FlowJo software (Treestar, Inc., CA, USA). For the co-culture experiment, CD4^+^ cells co-cultured with DAC-treated or –untreated DCs were incubated with 100 ng/ml phorbol 12-myristate 13-acetate (PMA) and 1 μg/ml ionomycin for 1 hour at 37 °C and then added 10 μg/ml brefeldin A (all from Sigma-Aldrich, MO, USA) for an additional 4 hours. The frequency of CD4^+^ IFN-γ^+^ and CD4^+^ IL-17A^+^ cells were detected by flow cytometry. Briefly, CD4^+^ cells were permeabilized by using the Intracellular Fixation & Permeabilization Set (eBioscience, CA, USA). The cells were subsequently stained with anti-human IL-17A-PE (eBioscience, CA, USA) and anti-human IFN-γ-FITC (eBioscience, CA, USA) for 30 minutes at 4 °C.

### Enzyme-linked immunosorbent assay (ELISA)

Human Duoset ELISA kits (R&D Systems, MN, USA) were used to detect the protein expression of interleukin(IL)-1β, IL-6, IL-17 and IFN-γ. The concentration of IL-23 and IL-12p70 was detected by the human IL-23 Ready-SET-GO kit and IL-12(p70) high sensitivity kit (eBioscience, CA, USA) in accordance with the manufacturer’s instructions.

### Statistical Analysis

All the results are shown as mean ± SEM. The SPSS 22.0 software (SPSS Inc, IL, USA) and GraphPad Prism 5 software (GraphPad Software, Inc., CA) were used to do the statistical analysis. The independent-sample t-test or paired-samples t-test was performed to analyze the data. One way-ANOVA with Bonferroni correction was used to analyze multiple comparison. The *p*-value less than 0.05 was taken for significantly different.

## Electronic supplementary material

Supplementary tables
