# Supplementary material for: Hypermethylation of Interferon Regulatory Factor 8 (IRF8) Confers Risk to Vogt-Koyanagi-Harada Disease
Source: Sci Rep. 2017 Apr 21;7:1007. doi: 10.1038/s41598-017-01249-7 (PMC5430771; doi:10.1038/s41598-017-01249-7)
Supplement: Supplementary file 1 — Supplementary tables [file 41598_2017_1249_MOESM1_ESM.doc]

**Hypermethylation of Interferon Regulatory Factor 8 (IRF8)** **Confers Risk to Vogt-Koyanagi-Harada Disease**

Yiguo Qiu^1^, Hongsong Yu^1^, Yunyun Zhu^1^, Zi Ye^1^, Jing Deng^1^, Wencheng Su^1^, Qingfeng Cao^1^, Gangxiang Yuan^1^, Aize Kijlstra^2^, Peizeng Yang^1^

^1^ The First Affiliated Hospital of Chongqing Medical University, Chongqing Key Laboratory of Ophthalmology, Chongqing Eye Institute, Chongqing, China

^2^ University Eye Clinic Maastricht, Maastricht, the Netherlands

**Supplementary** Table S1:

| **Table S1.** Protein level fold changes of inflammatory cytokines following DAC treatment | | |
| --- | --- | --- |
| group | cytokines | protein fold changes |
| Normal controls vs. VKH patients | IL-6 | 3.7 |
|  | IL-1β | 4.0 |
|  | IL-23 | 2.9 |
|  | IL-12p70 | 3.3 |
| VKH vs. VKH + DAC | IL-6 | 2.0 |
|  | IL-1β | 2.1 |
|  | IL-23 | 1.4 |
|  | IL-12p70 | 3.9 |

**Supplementary Tables S2**:

| **Table S2.** Basic information of inactive VKH patients enrolled in the study | | | |
| --- | --- | --- | --- |
| **Patient number** | **Age** | **Gender** | **Medications** |
| Case 1 | 55 | F | PDN: 5 mg, qd + CsA: 25 mg, bid |
| Case 2 | 24 | F | PDN: 5 mg, qd + CsA: 25 mg, bid |
| Case 3 | 28 | F | PDN: 5 mg, qd + CsA: 50 mg, qd |
| Case 4 | 45 | F | PDN: 15 mg, qd + CsA: 100 mg, qd |
| Case 5 | 30 | M | PDN: 15 mg, qd + CsA: 125 mg, qd |
| Case 6 | 46 | M | PDN: 20 mg, qd + CsA: 100 mg, qd |
| Case 7 | 37 | M | PDN: 5 mg, qod + CsA: 75 mg, qd |
| Case 8 | 32 | M | PDN: 15 mg, qd + CsA: 100 mg, qd |
| Case 9 | 36 | M | PDN: 20 mg, qd + CsA: 125 mg, qd |
| Case 10 | 27 | M | PDN: 20 mg, qd + CsA: 75 mg, bid |
| PDN: Prednisone; CsA: cyclosporin a | | | |
